# 7.K. Workshop: The implementation of ”Good or Best Practices”: what can we learn from each other?

**DOI:** 10.1093/eurpub/ckac129.442

**Published:** 2022-10-25

**Authors:** Organised by: RIVM (Netherlands), BZgA (Germany), Finnish Institute of Health and Welfare, Chair persons: Djoeke Dale Van (Netherlands), Christin Rossmann (Germany)

## Abstract

**Introduction:**

The value of evidence-informed development and implementation of public health measures and practices - often called “good” or “best practices” - has been widely acknowledged in order to effectively address public health challenges. Yet information on such measures and practices remains insufficiently accessible to practitioners, policy and decision-makers. On a national level, several European countries have developed “Best Practice Portals” in order to support the uptake, implementation and dissemination of acknowledged public health best practices or policies.

**Objective:**

Six countries in Europe with a national “portal” have recently compared the rationale, structure, and processes used by their respective national portals, gaining first insights into how development and implementation of best practices can be further supported from a national level. The workshop will present these first insights, as well as further promising approaches to facilitate access to best practices, such as innovative digital tools which local public health actors, policy and decision-makers may use to inform selection of best practices for their contexts.

**Methods:**

The workshop starts with three presentations to introduce and to illustrate the topic. In the second half of the workshop we ask for an active contribution of the participants. For interaction, the open space method will be used.

**Programme:**

1. Three presentations of 10 minutes with 1-3 questions:

• The presentation of the results of a survey in Poland assessing the decision-makers’ needs with regard to the implementation of best practices/evidence-informed policymaking

• A descriptive case comparison of six European program registries and the European Best Practice Portal, illustrating different approaches to support implementation of evidence-informed public health measures and practices

• Presentation of a digital tool to support evidence-informed planning and implementation of healthy and active environments by local public health actors

2. Discussion with the open space method (20 min)

In each of the four corners of one room, there is a flipchart with a moderator and a discussion question. Every participant is free to choose one of the flipcharts to discuss the topic with other participants who have chosen the same topic. The participant is free to go to another corner if he/she wants to join also other discussions. Discussion topics are:

• What are the advantages and limitations of national best practice portals in supporting the implementation of evidence-informed practices?

• What are other strategies to improve implementation and what makes them successful?

• How digital planning tool can be useful in evidence-informed planning and implementation??

• How can national portals collaborate with the EU Best practice portal?

3. Conclusion (10 min): every flip chart moderator reflects on their discussion, presenting 3 of the most important findings.

**Key messages:**

• National best/good practice portals have an important role to play in supporting the implementation of good/best practices.

• Different strategies should be employed to facilitate implementation of evidence-informed practices.

